# Antibacterial and antibiotic resistance modifying activity of the extracts from *allanblackia gabonensis, combretum molle* and *gladiolus quartinianus* against Gram-negative bacteria including multi-drug resistant phenotypes

**DOI:** 10.1186/s12906-015-0726-0

**Published:** 2015-06-30

**Authors:** Aimé G. Fankam, Jules R. Kuiate, Victor Kuete

**Affiliations:** Department of Biochemistry, Faculty of science, University of Dschang, P.O. Box 67, Dschang, Cameroon

**Keywords:** Efflux pumps, Extracts, Gram-negative bacteria, MDR bacteria, Resistance modulators

## Abstract

**Background:**

Bacterial resistance to antibiotics is becoming a serious problem worldwide. The discovery of new and effective antimicrobials and/or resistance modulators is necessary to tackle the spread of resistance or to reverse the multi-drug resistance. We investigated the antibacterial and antibiotic-resistance modifying activities of the methanol extracts from *Allanblackia gabonensis, Gladiolus quartinianus* and *Combretum molle* against 29 Gram-negative bacteria including multi-drug resistant (MDR) phenotypes.

**Methods:**

The broth microdilution method was used to determine the minimal inhibitory concentrations (MIC) and minimal bactericidal concentrations (MBC) of the samples meanwhile the standard phytochemical methods were used for the preliminary phytochemical screening of the plant extracts.

**Results:**

Phytochemical analysis showed the presence of alkaloids, flavonoids, phenols and tannins in all studied extracts. Other chemical classes of secondary metabolites were selectively presents. Extracts from *A. gabonensis* and *C. molle* displayed a broad spectrum of activity with MICs varying from 16 to 1024 μg/mL against about 72.41 % of the tested bacteria. The extract from the fruits of *A. gabonensis* had the best activity, with MIC values below 100 μg/mL on 37.9 % of tested bacteria. Percentages of antibiotic-modulating effects ranging from 67 to 100 % were observed against tested MDR bacteria when combining the leaves extract from *C. molle* (at MIC/2 and MIC/4) with chloramphenicol, kanamycin, streptomycin and tetracycline.

**Conclusion:**

The overall results of the present study provide information for the possible use of the studied plant, especially *Allanblackia gabonensis* and *Combretum molle* in the control of Gram-negative bacterial infections including MDR species as antibacterials as well as resistance modulators.

## Background

Infectious diseases caused by multi-drug resistant (MDR) Gram-negative bacteria are worldwide health concern, causing increasingly morbidity and mortality particularly in developing countries [1]. In Cameroon, previous studies showed high levels of resistance to commonly used antibiotics in Gram-negative bacilli [2]. Several reports also mentioned an increase in the hospital dissemination of bacterial strains specifically those expressing drug efflux mechanism [3, 4]. Against Gram-negative bacteria, the discovery of efflux pump inhibitors (EPIs) is an attractive strategy to combat MDR phenotypes [5]. EPI generally interact with specific efflux pump proteins to restore the susceptibility of MDR bacteria to antibiotics [6]. Medicinal plants constitute an important source of chemotherapeutic molecules, in regards to the chemical diversity found in several species [7, 8]. In recent years, some plants have been successfully evaluated for their direct antibacterial action, and for their antibiotic-modulation activity [9–12]. In the present work, we hypothesized that herbal medicines traditionally used for the treatment of infectious diseases could contain molecules acting as antibacterial and/or antibiotic-resistance modulators. This study was therefore designed to investigate the *in vitro* antibacterial and antibiotic-resistance modifying activities of the methanol extracts from *Allanblackia gabonensis* Pellegr. (Clusiaceae), *Gladiolus quartinianus* A. Rich (Iridaceae) and *Combretum molle* R. Br. ex G. Don (Combretaceae) against Gram-negative bacteria including multi-drug phenotypes. These plants are traditionally used to manage various ailments including bacterial related infections.

## Methods

### Plant materials and extraction

Medicinal plants used in this work were collected in different areas of Cameroon between January and April 2012. The plants were identified at the National Herbarium (Yaoundé, Cameroon), where voucher specimens were deposited under the reference numbers (Table 1). The air-dried and powdered plant material was weighed (300 g) and soaked in 1 L of methanol (MeOH) for 48 h at room temperature. The filtrate obtained through Whatman filter paper No. 1 was concentrated under reduced pressure in vacuum to obtain the crude extracts. All crude extracts were then kept at 4 °C until further uses.

**Table 1:** Plants used in the present study and evidence of their bioactivities

**Samples, family, and herbarium number** ^**a**^	**Traditional treatment**	**Area of plant collection**	**Known bioactive or potentially active compounds**	**Screened biological activities of the crude extracts and known bioactive compounds**
*Allanblackia gabonensis* Pellegr. (Clusiaceae); 17275SRF/Cam	Dysentery, cold, toothache [13]; pain, rheumatism, inflammations [14, 15].	Lebialem, South West region of Cameroon; (4°10′N 9°14′E/4.167°N 9.233°E)	Not reported	Crude extracts: Analgesic and anti-inlammatory effect of aqueous extract of the stem bark [14]; crude methanol fruits extracts (40 μg/mL) showed to inhibit growth of CCRF-CEM leukemia cells at about 50 % [16].
*Gladiolus quartinianus* A. Rich (Iridaceae); 17260/SRF/Cam	Infections of the skin, gut, urogenital system, and upper respiratory tract [17], gonorrhea, infectious conditions, constipation and dysentery [18].	Lebialem, South-West region of Cameroon; (4°10′N 9°14′E/4.167°N 9.233°E)	Not reported	Methanol crude extract was reported to possess moderate to significant anticancer activity (IC50: 29.60 to10.57 μg/mL) against drug-resistance cancer cell lines [16]
*Combretum molle* R. Br. ex G. Don (Combretaceae); 33311/ HNC	Fever, abdominal pains, convulsion, worm infections, human immunodeficiency virus (HIV)/acquiredimmune deficiency syndrome (AIDS) related infections [19]; hookworm, snake bite, leprosy, dysentery, general body swellings, arthritic and other inflammatory conditions, sterility, constipation [20, 21]; Parasitic, protozoan, infectious diseases [22], malaria [23]	University of Dschang, West region of Cameroon; (6°30′N 10°30′E/6.500°N 10.500°E)	Mollic acid glucoside [21]); β-D-glucopyranosyl 2α,3β,6β-trihydroxy-23-galloylolean-12-en-28-oate,combregenin, arjungenin, arjunglucoside I, and combreglucoside [24] .	Crude extracts were evaluated for: antibacterial activity [25–27]; antimycobacterial [28]; antifungal effects [29]; antimalarial [30]; anthelmintic activities [31]; anti-HIV by inhibition of ribonuclease-H [19]; Cytotoxic effects against T-24 bladder cancer cells [32]; Anti-inflammatory activity [24]; *in vitro* anticholinesterase and inhibitory effects on Rabbit Breathing [33]. Compounds: mollic acid glucoside (MAG) showed analgesic, anti-inflammatory properties in mice and rats [21], cardiovascular effect [34]; hypoglycaemic effect [35]; Punicalgin and CM-A, two isolated tannins were assessed for their anti-HIV activity against human immunodeficiency virus type 1 (HIV-1) and type 2 (HIV-2) [36].

^a^Plants were identified at the Cameroon National Herbarium (HNC)

### Chemicals for antibacterial assays

Eight commonly used antibiotics including tetracycline (TET), kanamycin (KAN), streptomycin (STR), ciprofloxacin (CIP), norfloxacin (NOR), chloramphenicol (CHL), ampicillin (AMP), erythromycin (ERY) (Sigma-Aldrich, St Quentin Fallavier, France) were used. The *p-*Iodonitrotetrazolium chloride 0.2 % (INT) and phenylalanine arginine β-naphthylamide (PAβN) (Sigma-Aldrich) were used as bacterial growth indicator and efflux pumps inhibitor respectively.

### Microorganisms and growth conditions

Pathogenic bacteria used in the study were Gram-negative bacteria including MDR isolates (Laboratory collection) and reference strains (American Type Culture Collection) of *Escherichia coli* (ATCC8739, ATCC10536, AG100, AG100A, AG100A_Tet_, AG102, MC4100 W3110)*, Enterobacter aerogenes* (ATCC13048, CM64, EA27, EA3, EA289, EA298, EA294)*, Klebsiella pneumoniae* (ATCC11296, KP55, KP63, K24, K2)*, Enterobacter cloacae* (ECCI69, BM47, BM67), *Pseudomonas aeruginosa* (PA01, PA124) and *Providencia stuartii* (ATCC29916, NEA16, PS2636, PS299645) were used. Their features were previously reported [37]. They were maintained at 4 °C and sub-cultured on a fresh appropriate Mueller Hinton Agar (MHA) for 24 h before any antibacterial test.

### Preliminary phytochemical investigation

The plant extracts were screened for the presence of major secondary metabolite classes such as alkaloids, anthocyanins, anthraquinones, flavonoids, phenols, saponins, sterols and triterpenes according to common phytochemical methods previously described [38]. The tests were based on visual observation of the change in color or formation of precipitate after the addition of specific reagents.

### Antibacterial assays

MICs and MBCs of the plant extracts and chloramphenicol were determined by microdilution method using rapid INT colorimetric assay [25, 39]. Briefly, the samples were first dissolved in 10 % Dimethyl-sulfoxide (DMSO)/Mueller Hinton Broth (MHB). The solution obtained was then added to MHB and serially diluted two fold (in a 96-well microplate). One hundred microliters of inoculum (1.5× 10^6^ CFU/mL) prepared in MHB were then added. The plates were covered with a sterile plate sealer and then agitated with a shaker to mix the contents of the wells and incubated at 37 °C for 18 h. The final concentration of DMSO was less than 2.5 %, and did not affect the microbial growth. Wells containing MHB, 100 μL of inoculum, and DMSO at a final concentration of 2.5 % served as the negative control. The MIC of each sample was detected after 18 h of incubation at 37 °C following addition of 40 μL INT (0.2 mg/mL) and incubation at 37 °C for 30 min. Viable bacteria reduced the yellow dye to a pink. The MIC was defined as the lowest sample concentration that prevented this change and that resulted in the complete inhibition of bacterial growth. The MBC of the sample was determined by sub-culturing 50 μL of the suspensions from the wells which did not show any growth after incubation during MIC assays to 150 μl of fresh broth, and re-incubated at 37 °C for 48 h before revelation. The MBC was defined as the lowest concentration of sample which completely inhibited the growth of bacteria [40]. Each assay was performed in three independent tests in triplicate. The samples were also tested in the presence of phenylalanine arginine β-naphthylamide (PAβN) at a final concentration of 20 μg/mL as previously described [41] on nine MDR bacteria. All assays were performed three time in duplicate.

### Antibiotic-modulation assay

To evaluate the antibiotic resistance modifying activity of the extracts, the MIC of antibiotic was determined in the presence or absence of the plant extracts. The 96-wells plate modulation method, as described by Stavri *et al.* [42] was used. Briefly, after serial dilutions of antibiotics (256–0.5 μg/mL), the plant extracts at their sub-inhibitory concentrations (MIC/2 and MIC/4; selected after preliminary study; Table 2), were added and inoculation was done. The MIC was determined as described above. Modulation factors (MF), calculated as MIC _Antibiotic alone_/MIC _Antibiotic alone + Extract_; was used to express the modulating or synergy effects of the plant extracts.

**Table 2:** MIC of antibiotics in combination with extracts at sub-inhibitory concentrations against *P. aeruginosa* PA124

**Plant extracts** ^**a**^	**Antibiotcs** ^**b**^								
		**CHL**	**AMP**	**ERY**	**KAN**	**STR**	**TET**	**CIP**	**NFX**
	ATB Alone	64	-	128	128	64	16	32	128
AGR	MIC/2	64(1)	- (NA)	128(1)	16**(2)**	32(**2**)	16(1)	32(1)	128(1)
	MIC/4	64(1)	- (NA)	128(1)	16(2)	64(1)	16(1)	32(1)	128(1)
	MIC/8	64(1)	- (NA)	128(1)	128(1)	64(1)	16(1)	32(1)	128(1)
	MIC/16	64(1)	- (NA)	128(1)	128(1)	64(1)	16(1)	32(1)	128(1)
AGB	MIC/2	64(1)	- (NA)	128(1)	128(1)	32**(2)**	8**(2)**	32(1)	128(1)
	MIC/4	64(1)	- (NA)	128(1)	128(1)	64(1)	16(1)	32(1)	128(1)
	MIC/8	64(1)	- (NA)	128(1)	128(1)	64(1)	16(1)	32(1)	128(1)
	MIC/16	64(1)	- (NA)	128(1)	128(1)	64(1)	16(1)	32(1)	128(1)
AGL	MIC/2	64(1)	- (NA)	128(1)	16**(2)**	64(1)	16(1)	32(1)	128(1)
	MIC/4	64(1)	- (NA)	128(1)	128(1)	64(1)	16(1)	32(1)	128(1)
	MIC/8	64(1)	- (NA)	128(1)	128(1)	64(1)	16(1)	32(1)	128(1)
	MIC/16	64(1)	- (NA)	128(1)	128(1)	64(1)	16(1)	32(1)	128(1)
**AGF**	**MIC/2**	32**(2)**	- (NA)	128(1)	64**(2)**	32**(2)**	16(1)	32(1)	128(1)
	**MIC/4**	64(1)	- (NA)	128(1)	64**(2)**	32**(2)**	16(1)	32(1)	128(1)
	MIC/8	64(1)	- (NA)	128(1)	64**(2**)	64(1)	16(1)	32(1)	128(1)
	MIC/16	64(1)	- (NA)	128(1)	64**(2)**	64(1)	16(1)	32(1)	128(1)
AGFl	MIC/2	64(1)	- (NA)	128(1)	64**(2)**	64(1)	8**(2)**	32(1)	128(1)
	MIC/4	64(1)	- (NA)	128(1)	64**(2)**	64(1)	16(1)	32(1)	128(1)
	MIC/8	64(1)	- (NA)	128(1)	64**(2)**	64(1)	16(1)	32(1)	128(1)
	MIC/16	64(1)	- (NA)	128(1)	128	64(1)	16(1)	32(1)	128(1)
GQW	MIC/2	64(1)	- (NA)	128(1)	64**(2)**	32**(2)**	16(1)	32(1)	128(1)
	MIC/4	64(1)	- (NA)	128(1)	64**(2)**	64(1)	16(1)	32(1)	128(1)
	MIC/8	64(1)	- (NA)	128(1)	128	64(1)	16(1)	32(1)	128(1)
	MIC/16	64(1)	- (NA)	256(0.5)	128	64(1)	16(1)	32(1)	128(1)
**CML**	**MIC/2**	32**(2)**	- (NA)	64**(2)**	64**(2)**	32**(2)**	4**(4)**	16**(2)**	16(**2)**
	**MIC/4**	32**(2)**	- (NA)	64**(2)**	64**(2)**	32**(2)**	4**(4)**	16**(2)**	16**(2)**
	MIC/8	64(1)	- (NA)	128(−)	64(**2)**	64(1)	8**(2)**	32(1)	128(1)
	MIC/16	641)	- (NA)	128(−)	64**(2)**	64(1)	8**(2)**	32(1)	128(1)

^**a**^ **: Plan extracts:** (AGFl *: Allanblackia gabonensis* Flowers, AGF : *Allanblackia gabonensis* Fruits, AGL: *Allanblackia gabonensis* Leaves*,* AGB: *Allanblackia gabonensis* Stem barks*,* AGR: *Allanblackia gabonensis Root* barks, GQW: *Gladiolus quartinianus* Whole plant*,* CML: *Combretum molle* Leaves); ^**b**^ *:* **Antibiotics (**TET: tetracycline, KAN: kanamycin, STR: streptomycin, ERY: erythromycin, CHL: chloramphenicol, NFX: norfloxacin, CIP: ciprofloxacin, AMP: ampicillin); − **:** MIC not detected at up to 256 μg/mL; **():** Modulation factor or gain of activity; NA: Not applicable, Values in bold represent the modulation factor ≥ 2, the selected extracts and its concentrations to be used on others MDR bacteria

## Results

### Phytochemical Screening of the plant extracts

The main classes of secondary metabolites for each extract were screened and the results are summarized in Table 3. Tannins, flavonoids, alkaloids and phenols were present in all tested extracts. Others classes of botanicals were selectively distributed in different plant extracts.

**Table 3:** Qualitative phytochemical composition of the plant extracts

**Plant samples**	**Phytochemical composition**								
	**Triterpenes**	**Flavonoids**	**Alkaloids**	**Anthraquinones**	**Phenols**	**Anthocyanines**	**Saponins**	**Tannins**	**Steroids**
***Allanblackia gabonensis***	-	+	*+*	+	+	+	_	*+*	-
	-	+	+	-	+	+	-	+	-
	+	+	+	+	+	+	+	+	-
	+	+	+	+	+	+	+	+	-
	+	+	+	-	+	+	+	+	-
***Gladiolus quartinianus***	+	+	+	**-**	+	+	-	+	+
***Combretum molle***	-	+	+	-	+	-	+	+	+

(+): present; (−): absent

### Antibacterial activity of the plant extracts

The results summarized in Table 4 show that all extracts were active on at least three of bacterial strains, with MIC values varying from 16 to 1024 μg/mL. Extracts from *Combretum molle* leave*s* (CML) and *Allanblackia gabonensis* displayed the most important spectrum of activity. Their inhibitory effects were observed on 72.41 % (27/29) for CML of the tested bacteria, 58.62 % (17/29) for leaves (AGL), 75.86 % (22/29) for flower (AGFl) and bark (AGB), 79.31 % (23/29) for bark and 86.20 % (25/29) for fruits (AGF) extracts from *Allanblackia gabonensis*. AGF was the best extract with MIC values below 100 μg/mL on 38 % (11/29) of the tested bacteria. CML mostly showed moderate activity with MIC values ranging from 128–512 μg/mL. The activity of CHL was comparable to that of plant extracts in certain cases. This was the case with AGF, AGFl and AGR against *K. pneumoniae* Kp55 (64 μg/mL); AGF against *K. pneumoniae* Kp53 (64 μg/mL), and AGFl against *P. stuartii* PS2636 (32 μg/mL). MICs values equal or above 1024 μg/mL were obtained with the extract from *G. quartinianus* (GQW). MBCs values were generally equal or below 1024 μg/mL (Table 4).

**Table 4:** Minimal inhibitory concentration (MIC) and minimal bactericidal (MBC) of the plant extracts and CHL on the studied bacterial species

**Bacteria used**	**Tested samples, MIC and MBC (μg/mL)**															
	***Allanblackia gabonensis***										***Gladiolus quartinianus*** (GQW)		***Combretum molle*** (CML)		**Chloramphenicol**	
	Flowers (AGFl)		Fruits (AGF)		Leave (AGL)		Stem barks (AGB)		Root barks (AGR)							
	MIC	MBC	MIC	MBC	MIC	MBC	MIC	MBC	MIC	MBC	MIC	MBC	MIC	MBC	MIC	MBC
***E. coli***																
ATCC8739	**64**	512	**16**	256	512	-	**64**	256	**64**	256	1024	-	256	1024	2	32
ATCC10536	**64**	512	**32**	256	512	1024	**32**	128	**64**	256	1024	-	512	512	1	32
AG100	-	-	1024		-	-	1024	-	512	-	-	-	512	1024	8	128
AG100A	128	512	**64**	256	512	512	128	512	128	512	-	-	512	1024	0.5	64
AG100A_Tet_	1024	-	512	-	-	-	1024	-	1024	-	-	-	1024	-	64	256
AG102	1024	-	1024	-	-	-	1024	-	1024	-	-	-	1024	-	32	>256
MC4100	256	512	128	1024	512	-	256	512	512	-	1024	-	256	1024	32	>256
W3110	256	-	256	512	512	1024	**64**	512	256	1024	-	-	512	1024	8	128
***E. aerogenes***																
ATCC13048	128	1024	**16**	128	256	1024	**32**	512	**32**	512	1024	-	1024	-	8	64
CM64	-	-	1024	-	-	-	-		-		-	-	1024	-	>256	>256
EA27	512	1024	128	512	512	-	128	512	128	512	-	-	1024	1024	64	256
EA3	1024	-	-	-	1024	-	512	-	512	-	-	-	1024	-	128	>256
EA289	1024	-	1024	-	-	-	-	-	1024	-	-	-	1024	-	128	>256
EA298	1024	-	-	-	-		-	-	-	-	-	-	1024	-	64	>256
EA294	128	256	**64**	512	256	1024	**64**	512	128	1024	1024	-	128	512	16	64
***E. cloacae***																
ECCI69	-	-	1024	-	-	-	-	-	1024	-	-	-	-	-	>256	>256
BM47	-	-	1024	-	-	-	1024	-	-	-	-	-	-	-	>256	>256
BM67	-	-	-	-	-	-	-	-	-	-	-	-	1024	-	>256	>256
***K. pneumoniae***																
ATCC11296	128	1024	**16**	128	256	1024	**32**	256	**32**	256	1024	-	512	1024	4	32
KP55	**64**	256	**64**	256	1024	-	256	1024	**64**	512	-	-	512	1024	64	256
KP63	128	256	**64**	256	256	1024	128	512	128	256	-	-	512	-	64	256
K24	-	-	1024		-		1024	-	1024	-	-	-	1024	-	32	256
K2	128	1024	**32**	128	512	1024	128	256	**64**	256	-	-	256	1024	16	256
***P. stuartii***																
ATCC29916	**64**	512	**16**	128	256	512	128	512	**64**	256	-	-	512	512	8	128
NEA16	1024	-	1024	-	-		-	-	-	-	-	-	1024	-	64	256
PS2636	**32**	512	**64**	128	512	-	256	256	128	512	-	-	512	-	32	256
PS299645	128	1024	128	-	128	512	256	512	128	512	-	-	512	512	32	256
***P. aeruginosa***																
PA01	512	1024	512	512	512		512	1024	512	-	1024	-	512	1024	32	256
PA124	-	-	-		-		-		-	-	-	-	1024	-	64	>256

**- - :** MIC and MBC not detected at up to 1024 μg/mL, Values in bold represent significant antibacterial activity of the plant extract

### Antibacterial activity of the extracts in the presence of an Efflux Pumps Inhibitors

In the present work, extracts were combined with PAβN; However, no significant increase of the activities of the tested plant extracts was generally observed. Only AGL showed 4 times decrease of MICs against *E. coli* AG102 and *E. cloacae* BM67. In contrast, PAβN significantly improved the activity of the reference drug, CHL (more than 16 times) on MDR bacteria used (Table 5).

**Table 5:** MIC of the samples in the absence and presence of PAβN on the selected MDR bacterial species

**MDR Bacteria used**	**Samples and MIC (μg/mL)**															
	***Allanblackia gabonensis***										***Gladiolus quartinianus*** (GQW)		***Combretum molle*** (CML)		**chloramphenico**l	
	Flowers (AGFl)		Fruits (AGF)		Leaves (AGL)		Stem bark (AGB)		Root barks (AGR)							
	Alone	+ PAβN	alone	+ PAβN	Alone	+ PAβN	alone	+ PAβN	Alone	+ PAβN	Alone	+ PAβN	Alone	+ PAβN	Alone	+ PAβN
*E. coli*																
AG100A_Tet_	1024	1024	1024	512	-	-	1024	1024	1024	1024	-	-	1024	1024	**64**	**≤2**
AG102	1024	1024	1024	512	**-**	**512**	1024	1024	124	1024	-	-	1024	1024	**32**	**≤2**
***E. aerogenes***																
CM64	-	-	1024	1024	-	-	-	-	-	-	-	-	1024	1024	**256**	**16**
EA289	1024	1024	1024	1024	-	1024	-	-	1024	1024	-	-	1024	512	**128**	**8**
***E. cloacae***																
ECCI69	-	-	1024	1024	-	-	-	-	-	-	-	-	-	-	>256	128
BM67	-	1024	-	-	**-**	**512**	-	-	-	-	-	-	1024	1024	**>256**	**32**
***K. pneumoniae***																
K24	-	1024	1024	1024	-	-	1024	1024	1024	1024	-	-	1024	1024	**32**	**2**
***P. stuartii***											-					
NEA16	-	-	1024	1024	-	-	-	1024	-	1024	-	-	1024	1024	**64**	**8**
***P. aeruginosa***																
PA124	-	-	-	-	-	-	-	-	-	-	-	-	1024	1024	**64**	**8**

### Antibiotic resistance modifying activities of the plant extracts

Tables 2, 6 and 7 highlights the potentiating effects of the extracts on the activity of eight commonly used antibiotics. The most important modulating effects were observed of association CML with aminoglycosides (kanamycin and streptomycin), the potentiation effects varying from 77.78 to 88.89 % and from 66.67 to 77.78 % at MIC/2 and MIC/4 respectively; and with tetracycline (100 % and 77.78 % at MIC/2 and MIC/4 respectively) (Table 6). The modulating effects also ranged between 50 to 67 %, with the extract from *A. gabonensis* fruits (AGF) when combined at (MIC/2) with the some antibiotics. At MIC/4, AGF showed synergy less than 50 % on the tested bacteria with all antibiotics (Table 7). The most significant increases of antibiotic activity in the presence of plant extracts were noted with the association of streptomycin and CML and AGF on *E. coli* AG100A_Tet,_ with more than 128 fold and 64-fold decreases of MIC respectively. No increase of activity was noted with ampicillin, a β-lactamine when it was combined with plant extracts.

**Table 6:** Resistance modulating effect of the methanol leaves extract from *Combretum molle* at its sub-inhibitory concentrations on selected

**Antibiotics** ^**a**^	**Extract concentrations**	**MDR Bacteria used**									**% Modulating effect** ^**b**^
		***E. coli***			***E. aerogenes***			***K. pneumoniae***	***P. stuartii***	***P. aeruginosa***	
		**AG100**	**AG102**	**AG100**_**Tet**_	**CM64**	**EA289**	**EA298**	**K24**	**NEA16**	**PA124**	
AMP	0	-	-	-	-	-	-	-	-	-	
	MIC/2	-(NA)	-(NA)	-(NA)	-(NA)	-(NA)	-(NA)	-(NA)	-(NA)	-(NA)	0
	MIC/4	-(NA)	-(NA)	-(NA)	-(NA)	-(NA)	-(NA)	-(NA)	-(NA)	-(NA)	0
CHL	0	8	32	64	256	128	64	64	64	64	
	MIC/2	8(1)	16 (**2**)	32(**2**)	256(1)	64(**2**)	8(**8**)	64(1)	16(**4**)	32(**2**)	**66.65**
	MIC/4	8(1)	32(1)	64(1)	256(1)	64(**2**)	32(**2**)	64(1)	32(**2**)	32(**2**)	44.44
CIP	0	≤0.5	≤0.5	128	64	128	1	128	1	32	
	MIC/2	≤0.5(NA)	≤0.5	64(**2**)	64(1)	128(1)	1(1)	64(**2**)	1(1)	16(**2**)	33.33
	MIC/4	≤0.5(NA)	≤0.5	64(**2**)	64(1)	128(1)	1(1)	64(**2**)	1(1)	16(**2**)	33.33
NFX	0	≤0.5	≤0.5	256	-	128	8	-	4	128	
	MIC/2	≤0.5(NA)	≤0.5	128(**2**)	64(**>4**)	128(1)	8(1)	-(NA)	4(1)	64(**2**)	33.33
	MIC/4	≤0.5(NA)	≤0.5	256(1)	64(**>4**)	128(1)	8(1)	-(NA)	4(1)	64(**2**)	22.22
**KAN**	0	64	4	8	2	16	32	4	16	128	
	MIC/2	4(**16**)	4(1)	4(**2**)	≤0.5(**>4**)	2(**8**)	32(1)	2(**2**)	4(**4**)	64(**2**)	**77.78**
	MIC/4	8(**8**)	4(1)	4(**2**)	1(**2**)	4(**4**)	32(1)	2(**2**)	8(**2**)	64(**2**)	**77.78**
**STR**	0	>64	-	-	4	16	32	4	32	64	
	MIC/2	>64(NA)	256 (**≥2)**	4(**>64**)	1(**4**)	4(**4**)	16(**2**)	1(**4**)	8(**4**)	32(**2**)	**88.89**
	MIC/4	>64(NA)	-	128(**>2**)	2(**2**)	4(**4**)	32(1)	2(**2**)	16(**2**)	32(**2**)	**66.67**
ERY	0	32	64	**-**	-	64	16	128	16	128	
	MIC/2	8(**4**)	64(1)	256(>1)	-(NA)	64(1)	4(**4**)	128(1)	4(**4**)	64(**2**)	44.44
	MIC/4	8(**4**)	64(1)	-(NA)	-(NA)	64(1)	16(1)	128(1)	8(**2**)	128(1)	22.22
**TET**	0	2	8	32	16	32	4	16	4	16	
	MIC/2	≤0.5(**>4**)	2(**4**)	8(**4**)	4(**4**)	8(**4**)	2(**2**)	8(**2**)	2(**2**)	4(**4**)	**100**
	MIC/4	1(**2**)	8(1)	32(1)	8(**2**)	8(**4**)	2(**2**)	8(**2**)	2(**2**)	4(**4**)	**77.78**

^**a**^ **: Antibiotics** (TET: tetracycline, KAN: kanamycin, STR: streptomycin, ERY: erythromycin, CHL: chloramphenicol, NFX: norfloxacin, CIP: ciprofloxacin, AMP: ampicillin; **- :** MIC not detected at up to 256 μg/mL**; ():** Modulation factor or gain of activity; ^**b**^ **:** Percentage of Antibiotic’s modulation Activity by the Plant extracts; Indications in bold represent the modulation factor ≥ 2, antibiotics having a percentage of modulating effect greather than 50 %; NA: Not applicable

**Table 7:** Resistance modulating effect of the Fruits methanol extract from *Allanblackia gabonensis* at its sub-inhibitory concentrations on selected MDR bacteria

**Antibiotics** ^a^	**Extract concentrations**	**MDR Bacteria used**									**% Modulating effect** ^**b**^
		***E. coli***			***E. aerogenes***			***K. pneumoniae***	***P. stuartii***	***P. aeruginosa***	
		**AG100**	**AG102**	**AG100A**_**Tet**_	**CM64**	**EA289**	**EA298**	**K24**	**NEA16**	**PA124**	
AMP	0	-	-	-	-	-	-	-	-	-	
	MIC/2	-(NA)	-(NA)	-(NA)	-(NA)	-(NA)	-(NA)	-(NA)	-(NA)	-(NA)	0
	MIC/4	-(NA)	-(NA)	-(NA)	-(NA)	-(NA)	-(NA)	-(NA)	-(NA)	-(NA)	0
CHL	0	8	32	64	256	128	64	64	64	64	
	MIC/2	8(1)	32(1)	64(1)	256(1)	128(1)	32**(2)**	32(**2**)	64(1)	32(**2**)	33.33
	MIC/4	8(1)	32(1)	64(1)	256(1)	128(1)	64(1)	64(1)	64(1)	64(1)	0
CIP	0	≤0.5	≤0.5	128	64	128	1	128	1	32	
	MIC/2	≤0.5(NA)	≤0.5(NA)	64(**2**)	32**(2)**	128(1)	2(0.5)	128(1)	1(1)	32(1)	33.33
	MIC/4	≤0.5(NA)	≤0.5(NA)	64(**2**)	64(1)	128(1)	2(0.5)	128(1)	1(1)	32(1)	11.11
NFX	0	≤0.5	≤0.5	256	-	128	8	-	4	128	
	MIC/2	1(≤0.5)	≤0.5(NA)	256(1)	128(**>2**)	128(1)	8(1)	-(NA)	4(1)	128(1)	11.11
	MIC/4	1(≤0.5)	≤0.5(NA)	256(1)	-(NA)	128(1)	8(1)	-(NA)	4(1)	128(1)	0
**KAN**	0	64	4	8	2	16	32	4	16	128	
	MIC/2	2(**32**)	4(1)	2(**4**)	≤0.5(**≥4**)	1(**16**)	32(1)	2(**2**)	16(1)	64(**2**)	**66.67**
	MIC/4	4(**16**)	4(1)	4(**2**)	2(1)	4(**4**)	32(1)	4(1)	16(1)	64(**2**)	44.44
**STR**	0	>64	-	-	4	16	32	4	32	64	
	MIC/2	>64(NA)	-(NA)	2(**>128**)	2(**2**)	2(**8**)	32(1)	4(1)	16(**2**)	32(**2)**	55.56
	MIC/4	>64(NA)	-(NA)	8(**>32**)	2(**2**)	4(**4**)	32(1)	4(1)	32(1)	32**(2)**	44.44
ERY	0	32	64	-	-	64	16	128	16	128	
	MIC/2	16(**2**)	64(1)	256(>1)	-(NA)	64(1)	16(1)	128(1)	16(1)	128(1)	11.11
	MIC/4	16(**2**)	64(1)	-(NA)	-(NA)	64(1)	16(1)	128(1)	16(1)	128(1)	0
**TET**	0	2	8	32	16	32	4	16	4	16	
	MIC/2	1(**2**)	4**(2)**	8(**4**)	4(**4**)	4(**8**)	4(1)	16(1)	2(**2**)	16(1)	**66.67**
	MIC/4	1(**2**)	8(1)	8(**4)**	4(**4**)	8(**4**)	4(1)	16(1)	4(1)	16(1)	44.44

^**a**^ **: Antibiotics** (TET: tetracycline, KAN: kanamycin, STR: streptomycin, ERY: erythromycin, CHL: chloramphenicol, NFX: norfloxacin, CIP: ciprofloxacin, AMP: ampicillin; **- :** MIC not detected at up to 256 μg/mL**; ():** Modulation factor or gain of activity; ^**b**^ **:** Percentage of antibiotic’s modulation activity by the Plant extracts; Indications in bold represent the modulation factor ≥ 2 and antibiotics having a percentage of modulating effect greather than 50 %; NA: Not applicable

## Discussion

Medicinal plants are potential source of antimicrobial agents used in the treatment of infectious diseases [43, 44]. According to Kuete *et al.* [45, 46], the antibacterial activity of a plant extract is considered significant when the MICs are below 100 μg/mL, moderate when 100 ≤ MIC ≤ 625 μg/mL and weak when MIC are above 625 μg/mL. Consequently, the antibacterial activities of the tested extracts particularly those from *A. gabonensis* (AGF, AGR, AGB and AGFl) and *C. molle* (CML) were generally moderated (Table 4). Significant activities were recorded with AGF, AGR, AGB, and AGFl respectively on 37.93 %, 24.14 %, 20.70 % and 17.24 %. This highlights the good antibacterial potential of the tested extracts. The overall activity recorded with most of the studied extracts could be considered significant, especially those from *A. gabonensis* and *C. molle* [47]*.* When analyzing carefully the MIC and MBC results for the extract, it can be noted that MBC ≤ 4 were obtained with these samples on most of the tested bacterial species, suggesting their killing effects [48].

PAβN is a potent inhibitor of RND systems like AcrAB-TolC in Enterobacteriaceae or MexAB-OprM in *P. aeruginosa* used in the present work [49, 50]; The activity observed when chloramphenicol was tested in the presence of PAβN increased significantly, confirming that the tested bacteria are good models of efflux pumps-expressing bacteria.

Reversal of multi-drug resistance appears today as another attempt to mitigate the spread of resistance in bacteria. In recent years, many plants extracts and secondary metabolites have been evaluated as modulators of the antibiotic activity in efflux pumps in MDR bacteria [9–11, 37, 51–54]. Herein, we demonstrated that a beneficial effect of the combination of the extracts from the leaves of *C. molle* (CML) and fruits of *A. gabonensis* (AGF) with CHL, KAN and STR could be achieved. Synergistic or modulating effects of the plant extracts particularly *C. molle* extract with antibiotics were noted on more than 70 % of the tested MDR bacteria, suggesting that some of their constituents can act as efflux pump inhibitors [51]. These constituents might act by blocking the efflux pumps located in the cell membrane in the tested bacteria, preventing the extrusion of antibiotics in the cytoplasm and therefore restoring their activity as observed in this study [55, 56]. This is the first time to report the potential of the studied extracts, particularly those from the leaves of *C. molle* (CML) and the fruits of *A. gabonensis* (AGF) to reverse antibiotic resistance in MDR bacteria.

## Conclusion

This study provides informative data on the antimicrobial potential of the tested plant extracts and suggests that extracts from *Allanblackia gabonensis* could be a source of natural antibacterial products whilst *Combretum molle* leaves extract could contain both antibacterial substances and antibiotic-modulation agents. These data indicate that these plants can be used to fight bacterial infections and especially those involving MDR phenotypes.

## References

[1] Sharma R, Sharma CL, Kapoor B (2005). Antibacterial resistance: current problems and possible solutions. Indian J Med Res.

[2] Gangoue-Pieboji J, Bedenic B, Koulla-Shiro S, Randegger C, Adiogo D, Ngassam P (2005). Extended-spectrum-beta-lactamase-producing Enterobacteriaceae in Yaounde, Cameroon. J Clin Microbiol.

[3] Davin-Regli A, Bolla JM, James CE, Lavigne JP, Chevalier J, Garnotel E (2008). Membrane permeability and regulation of drug “influx and efflux” in enterobacterial pathogens. Curr Drug Targets.

[4] Pagès JM, Alibert-Franco S, Mahamoud A, Bolla JM, Davin-Regli A, Chevalier J (2010). Efflux pumps of gram-negative bacteria, a new target for new molecules. Curr Top Med Chem.

[5] Nikaido H, Pagès JM (2012). Broad specificity efflux pumps and their role in multidrug resistance of gram negative bacteria. FEMS Microbiol Rev.

[6] Lomovskaya O, Watkins W (2001). “Inhibition of efflux pumps as a novel approach to combat drug resistance in bacteria.”. J Mol Microbiol Biotechnol.

[7] Cowan MM (1999). Plant products as antimicrobial agents. Clin Microbiol Rev.

[8] Newman JD, Cragg GM (2012). Natural products as sources of new drugs over the 30 years from 1981 to 2010. J Nat Prod.

[9] Stermitz F, Lorenz P, Tawara JN, Zenewicz LA, Lewis K (2000). Synergism in a medicinal plant: antimicrobial action of berberine potentiated by S-methoxyhydrocarpin-a multidrug pump inhibitor. Proc Natl Acad Sci.

[10] Gibbons S (2005). Plants as a source of bacterial resistance modulators and anti-infective agents. Phytochem Rev.

[11] Bama SS, Kingsley SJ, Anan S, Bama P (2012). Antibacterial activity of different phytochemical extracts from the leaves of T. procumbens: Identification and mode of action of the terpeniod compounds as antibacterials. Int J Pharm Pharma Sci.

[12] Fankam A, Kuiate J-R, Kuete V (2014). Antibacterial activities of *Beilschmiedia obscura* and six other Cameroonian medicinal plants against multi-drug resistant Gram-negative phenotypes. BMC Complement Altern Med.

[13] Raponda-Walker A, Sillans R (1976). Les plantes utiles du Gabon.

[14] Ymele EV, Dongmo AB, Dimo T (2013). “Analgesic and anti-inlammatory efect of aqueous extract of the stem bark of *Allanblackia gabonensis* (Guttiferae)”. Inflammopharmacology.

[15] Kuete V, Efferth T (2011). Pharmacogenomics of Cameroonian traditional herbal medicine for cancer therapy. J Ethnopharmacol.

[16] Kuete V, Fankam AG, Wiench B, Efferth T (2013). Cytotoxicity and modes of action of the methanol extracts of six cameroonian medicinal plants against multidrug-resistant tumor cells. Evid Based Complement Alternat Med.

[17] Nguedia JCA, Etoa FX, Beng VP, Lontsi D, Kuete V, Moyou RS (2004). Anti-candidal property and acute toxicity of *Gladiolus gregasius* Baker (Iridaceae). Pharm Méd Trad Afr.

[18] Ameh SJ, Obodozie OO, Olorunfemi PO, Okoliko IE, Ochekpe NA (2011). Potentials of *Gladiolus* corms as an antimicrobial agent in food processing and traditional medicine. J Microbiol Antimicrob.

[19] Bessong PO, Obi CL, Igumbor E, Andreola M-L, Litvak S (2004). *In vitro* activity of three selected South African medicinal plants against human immunodeficiency virus type-1 reverse transcriptase. Afr J Biotechnol.

[20] Fyhrquist P, Mwasumbi L, Haeggstro CA, Vuorela H, Hiltunen R, Vuorela P (2002). Ethnobotanical and antimicrobial investigation on some species of *Terminalia* and *Combretum* (Combretaceae) growing in Tanzania. J Ethnopharmacol.

[21] Ojewole JAO (2008). Analgesic and anti-inflammatory effects of mollic acid glucoside, a 1a- hydroxycycloartenoid saponin extractive from *Combretum molle* R. Br. ex G. Don (Combretaceae) leaf. Phytother Res.

[22] Bussmann RW, Gilbreath GG, Soilo J, Lutura M, Lutuluo R, Kunguru K (2006). Plant use of the Massai of Sekenani Valley, Massai Mara, Kenya. J Ethnobiol Ethnomed.

[23] Grønhaug TE, Glæserud S, Skogsrud M, Ballo N, Bah S, Diallo D (2008). Ethnopharmacological survey of six medicinal plants from Mali, West Africa. J Ethnobiol Ethnomed.

[24] Ponou BK, Barboni L, Teponno RB, Mbiantcha M, Nguelefack TB, Park HJ (2008). Polyhydroxyoleanane-type triterpenoids from *Combretum molle* and their anti-inflammatory activity. Phytochem Lett.

[25] Eloff JN (1998). A sensitive and quick microplate method to determine the minimal inhibitory concentration of plant extracts for bacteria. Planta Med.

[26] Njume C, Afolayan AJ, Samie A, Ndip RN (2011). Inhibitory and bactericidal potential of crude acetone extracts of *combretum molle* (combretaceae) on drug-resistant strains of *Helicobacter pylori*. J Health Popul Nutr.

[27] Nyenje ME, Ndip RN (2012). Bioactivity of the acetone extract of the stem bark of *Combretum molle* on selected bacterial pathogens: Preliminary phytochemical screening. J Med Plants Res.

[28] Asres K, Bucar F, Edelsbrunner S, Kartnig T, Höger G, Thiel W (2001). Investigations on antimycobacterial activity of some Ethiopian medicinal plants. Phytother Res.

[29] Masoko P, Picard J, Eloff JN (2007). The antifungal activity of twenty-four Southern African Combretum species (Combretaceae). South Afr J Bot.

[30] Asres K, Balcha F (1998). Phytochemical screening and *in vitro* antimalarial activity of the stem bark of *Combretum molle* R. Br ex G Don Ethiopian Pharm J.

[31] Ademola IO, Eloff JN (2010). *In vitro* anthelmintic activity of *Combretum molle* (R. Br. ex G. Don) (Combretaceae) against *Haemonchus contortus* ova and larvae. Vet Parasitol.

[32] Fyhrquist P, Mwasumbi L, Vuorela P, Vuorela H, Hiltunen R, Murphy C (2006). Preliminary antiproliferative effects of some species of *Terminalia*, *Combretum* and *Pteleopsis* collected in Tanzania on some human cancer cell lines. Fitoterapia.

[33] Yéo D, Koffi E, Bidié AP, Tako NA, Bahi C, Méité S (2010). *In vitro* anticholinesterase and inhibitory effects of the aqueous extract of *Combretum molle* (combretaceae) leaf on rabbit breathing. Trop J Pharm Res.

[34] Ojewole JAO (2008). Cardiovascular effects of mollic acid glucoside, a 1alpha-hydroxycycloartenoid saponin extractive from *Combretum molle* R Br. ex G. Don (Combretaceae) leaf. Cardiovasc J Afr.

[35] Ojewole JAO, Adewole SO (2009). Hypoglycaemic effect of mollic acid glucoside, a 1a-hydroxycycloartenoid saponin extractive from *Combretum molle* R. Br. ex G. Don (Combretaceae) leaf, in rodents. J Nat Med.

[36] Asres K, Bucar F (2005). Anti-HIV activity against immunodeficiency virus type 1 (HIV-I) and type II (HIV-II) of compounds isolated from the stem bark of *Combretum molle*. Ethiop Med J.

[37] Fankam A, Kuete V, Voukeng KI, Kuiate J-R, Pages J-M (2011). Antibacterial activities of selected Cameroonian spices and their synergistic effects with antibiotics against multidrug-resistant phenotypes. BMC Complement Altern Med.

[38] Harbone JB (1973). Phytochemical methods: a guide to modern techniques of plant analysis.

[39] Mativandlela SPN, Lall N, Meyer JJM (2006). Antibacterial, antifungal and antitubercular activity of *Pelargonium reniforme* (CURT) and *Pelargonium sidoides* (DC) (Geraniaceae) root extracts. S Afr J Bot.

[40] Zgoda JR, Porter JR (2001). A convenient microdilution method screening natural products against bacteria and fungi. Pharmaceut Biol.

[41] Ghisalberti D, Masi M, Pagès J-M, Chevalier J (2005). Chloramphenicol and expression of multidrug efflux pump in *Enterobacter aerogenes*. Biochem Biophys Res Commun.

[42] Stavri M, Piddock LJV, Gibbons S (2007). Bacterial efflux pumps 1inhibitors from natural sources. J Antimicrob Chemother.

[43] Butler MS, Buss AD (2006). Natural products—the future scaffolds for novel antibiotics?. Biochem Pharmacol.

[44] Kuete V, Kuete V (2013). Medicinal Plant Research in *Africa*. Pharmacology and chemistry.

[45] Rios JL, Recio MC (2005). Medicinal plants and antimicrobial activity. J Ethnopharmacol.

[46] Kuete V (2010). Potential of cameroonian plants and derived products against microbial infections: a review. Planta Med.

[47] Simões M, Bennett RN, Rosa EA (2009). Understanding antimicrobial activities of phytochemicals against multidrug resistant bacteria and biofilms. Nat Prod Rep.

[48] Carbonnelle B, Denis F, Marmonier A, Pinon G, Vague R (1987). Bactériologie médicale: Techniques usuelles.

[49] Lomovskaya O, Bostian KA (2006). Practical applications and feasibility of efflux pump inhibitors in the clinic–a vision for applied use. Biochem Pharmacol.

[50] Lorenzi V, Muselli A, Bernardini AF, Berti L, Pagès JM, Amaral L (2009). Geraniol restores antibiotic activities against multidrug-resistant isolate from Gram-negative species. Antimicrob Agents Chemother.

[51] Braga LC, Leite AAM, Xavier KGS, Takahashi JA, Bemquerer MP, Chartone-Souza E (2005). Synergic interaction between pomegranate extract and antibiotics against *Staphylococcus aureus*. Can J Microbiol.

[52] Figueredo FG, Ferreira EO, Lucena BFF, Torres CMG, Lucetti DL, Lucetti ECP (2013). Modulation of the Antibiotic Activity by Extracts from Amburana cearensis A. C. Smith and Anadenanthera macrocarpa (Benth.) Brenan. Biomed Res Int.

[53] Tankeo SB, Lacmata ST, Noumedem JAK, Dzoyem JP, Kuiate JR, Kuete V (2014). Antibacterial and antibiotic-potentiation activities of some Cameroonian food plants against multi-drug resistant gram-negative bacteria. Chin J Integr Med.

[54] Bolla J, Alibert-Franco S, Handzlik J, Chevalier J, Mahamoud A, Boyer G (2011). Strategies for bypassing the membrane barrier in multidrug resistant Gram-negative bacteria. FEBS Lett.

[55] Kuete V, Alibert-Franco S, Eyong KO, Ngameni B, Folefoc GN, Nguemeving JR (2011). Natural products against bacteria expressing multidrug resistant phenotype. Int J Antimicrob Ag.

[56] Kuete V, Ngameni B, Tangmouo JG, Bolla J-M, Alibert-Franco S, Ngadjui BT (2010). Efflux pumps are involved in the Gram negative bacterial defense against isobavachalcone and diospyrone, two natural products. Antimicrob Ag Chemother.

